# Biomechanical assessment of various punching techniques

**DOI:** 10.1007/s00414-020-02440-8

**Published:** 2020-10-14

**Authors:** Jiri Adamec, Peter Hofer, Stefan Pittner, Fabio Monticelli, Matthias Graw, Jutta Schöpfer

**Affiliations:** 1 Institute of Legal Medicine, Ludwig-Maximilian-University, Nussbaumst. 26, 80336 Munich, Germany; 2 Department of Forensic Medicine, Paris-Lodron University of Salzburg, Ignaz Harrer St. 79, 5020 Salzburg, Austria

**Keywords:** Punch, Impulse, Fist, Open hand, Forensic biomechanics

## Abstract

Punches without the use of instruments/objects are a common type of body violence and as such a frequent subject of medicolegal analyses. The assessment of the injuries occurred as well as of the potential of the assault to produce severe body harm is based on objective traces (especially the documented injuries of both parties involved) as well as the—often divergent—descriptions of the event. Quantitative data regarding the punching characteristics that could be used for the assessment are rare and originate mostly in sports science. The aim of this study was to provide physical data enabling/facilitating the assessment of various punching techniques. A total of 50 volunteers took part in our study (29 males and 21 females) and performed severe punches with the fist, with the small finger edge of the hand (karate chop), and with the open hand with both the dominant and the non-dominant hands in randomized order. The strikes were performed on a boxing pad attached to a KISTLER force plate (sampling frequency 10,000 Hz) mounted on a vertical wall. The punching velocity was defined as the hand velocity over the last 10 cm prior to the contact to the pad and ascertained by using a high-speed camera (2000 Hz). Apart from the strike velocity, the maximum force, the impulse (the integral of the force-time curve), the impact duration, and the effective mass of the punch (the ratio between the impulse and the strike velocity) were measured/calculated. The results show a various degree of dependence of the physical parameters of the strikes on the punching technique, gender, hand used, body weight, and other factors. On the other hand, a high degree of variability was observed that is likely attributable to individual punching capabilities. In a follow-up study, we plan to compare the “ordinary” persons with highly trained (boxers etc.) individuals. Even though the results must be interpreted with great caution and a direct transfer of the quantitative parameters to real-world situations is in general terms not possible, the study offers valuable insights and a solid basis for a qualified forensic medical/biomechanical assessment.

## Introduction

Punches are a common kind of body violence and are being assessed on a regular basis in expert witness testimonies. Apart from the correspondence between the asserted assault and the documented injuries, often questions are raised regarding the punch intensity (both the actual and the hypothetically possible for the involved individual and the particular kind of assault) and other biomechanical aspects regarding the specific act of violence (oftentimes with significant differences among the participants and/or witnesses). Considering any rank order of violent acts, there seems to be a general agreement that a strike with the palm is less violent than a fist punch [1], though sufficiently objectifiable biomechanical data to support or dismiss this opinion are missing. However, in the current literature, there are also (mostly) individual cases in which comparatively serious injuries are said to have occurred as a result of blows with the open hand so that such a statement must at least be critically questioned [2, 3].

Although there is some knowledge regarding punching capabilities in different settings, mostly related to sports and/or martial arts with punch intensity measured by different methods [4–9], to our knowledge there is no comprehensive study concentrating on forensic aspects of various kinds of strikes and punches with intraindividual and interindividual comparisons.

This study aimed to obtain a data basis enabling to characterize various aspects of punching performance for both the dominant and non-dominant hands, for men and women, and for various kinds of punching techniques for the use in legal medical assessment.

## Methods

A total number of 50 volunteers participated in the study (29 males, 21 females). First, each subject was explained the objective and the procedure and signed an informed consent prior to the measurement. The study has been approved by the ethical committee of the Ludwig-Maximilians-University (LMU) Munich. Individuals with health issues regarding one or both arms as well as persons that experienced relevant (> 1 year regularly) “punching” training (boxing, kickboxing, karate, martial arts, etc.) were excluded. The main anthropometric characteristics of the volunteers are summarized in Table 1.

**Table 1:** The characteristics of the subjects

	Male	Female	All
Number of subjects	29	21	50
Age (mean; min/max) [year]	38; 21/58	29; 21/44	34; 21/58
Body mass (mean; min/max) [kg]	87; 56/125	61; 46/82	76; 46/125
Body length (mean; min/max) [cm]	180; 165/196	167; 150/181	174; 150/196
Handedness (right/left)	28/1	20/1	48/2

**Fig. 1:**
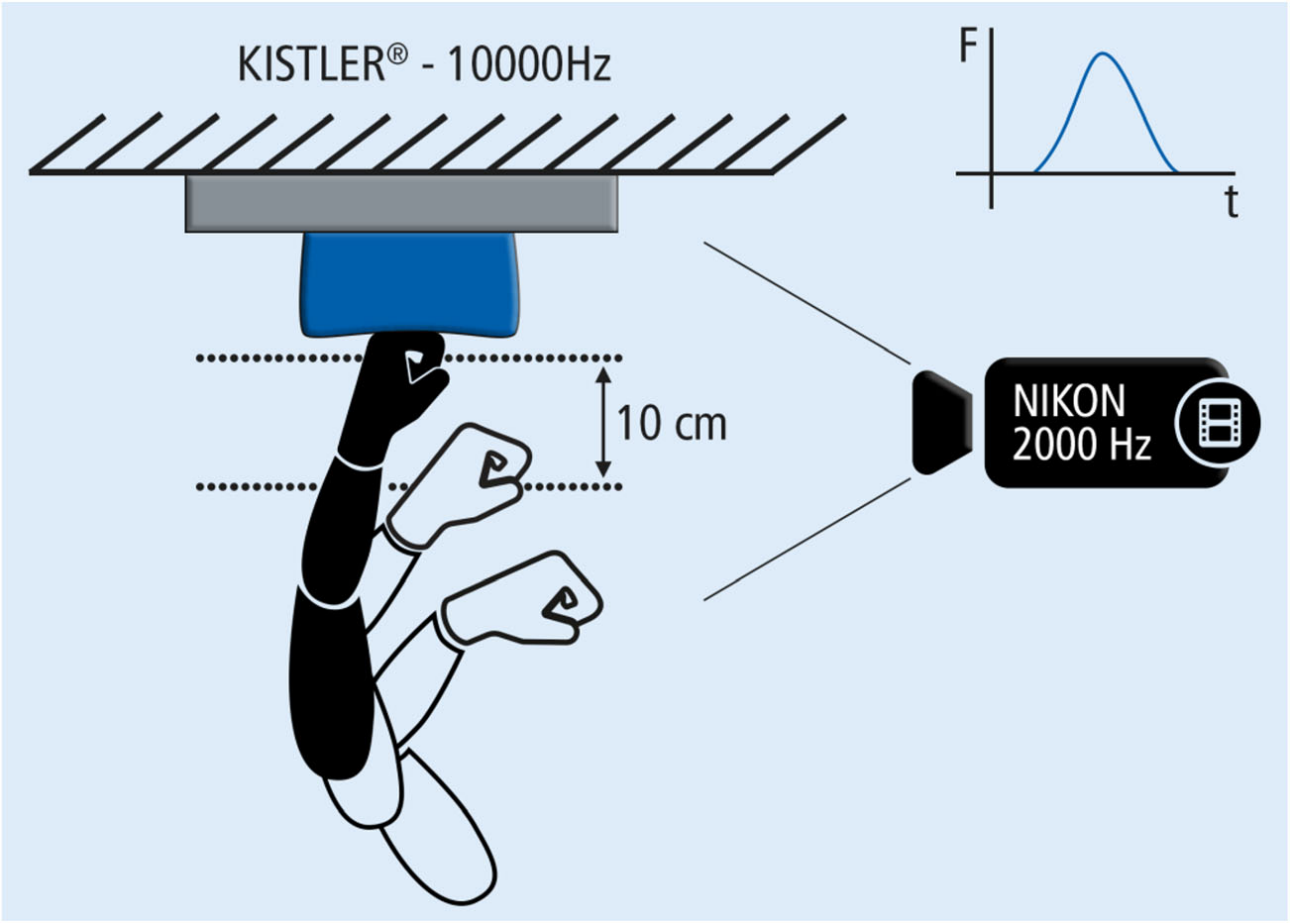
The measurement setup

The volunteers performed punches of three kinds—a fist punch (a punch with the knuckles of the clenched hand), a karate chop (a punch with the small finger edge of the hand, sometimes referred to as knife hand strike), and a palm strike (a punch with the palm aka a powerful slap). There were no precise instructions as how to perform the punching (for example, whether the chop should be performed as a forehand or as a backhand strike, whether the fingers should be held straight or flexed during the karate chop) and the volunteers were free to adopt any body position they liked before each punch. As a target, a punching pad (40 × 20 × 11 cm) was fastened on a KISTLER force plate (Type 9286 B, used with the Bioware software; sampling rate 10,000 Hz). In the course of the measurement session with each volunteer, three strikes were performed of every kind both with the dominant and the non-dominant hands. From the three measurements of the same kind, the one yielding the highest impulse (obtained by the integration of the force-time curve) was selected for further analysis. The last phase of the punch was recorded by a high-speed camera (Olympus® i-Speed 3 with the Nikon®-Lens AFNikkor 50 mm f/1, 8D; recording frequency 2000 Hz) and by measuring the time necessary for the hand to cover the last 10 cm before the impact and the velocity of the strike was calculated. The measurement setup is illustrated in Fig. 1. The hand motion was tracked manually in the video; a set of points was marked on the hand prior to the measurement in order to enhance the tracking process.

After a warm-up of each volunteer, the length of which was individual (until one felt comfortable with the setup and with him/her performing all kinds of punches with both hands), a total number of 18 trials were performed (three punches of three kinds for both hands) in a randomized order to eliminate possible learning and/or fatigue effects.

Since many (14 out of 30) of the measured/calculated impact parameters of various punch types turned out not to be normally distributed (the Shapiro test was used to test the normality of distribution), the Wilcoxon test was used to test the difference between the paired values for the dominant and non-dominant hands.

Accordingly, the Mann-Whitney test was used to test the differences between male and female subsamples.

Finally, the Friedman test was used to assess differences among the parameters of the three different punching techniques for the whole group. Post hoc tests (applicable only in case of significant Friedman test results) were performed according to the conservative Nemenyi method.

For all statistical analyses, a significance level of 0.05 was selected.

## Results

The basic characteristics of the measured/computed punch parameters are summarized in Tables 2 and 3 for the whole sample and for male and female volunteers, respectively. In both tables, significantly different parameters are italicized.

**Table 2:** The overview of punch parameters of the whole sample (*n* = 50). In 5 volunteers, the velocity of the karate chop with the dominant hand could not be measured; thus, *n* = 45 for the parameters velocity and effective mass; the corresponding values are marked by an asterisk. Italicized values are the mean values of parameters that differed significantly between the dominant and the non-dominant hands as indicated by the Wilcoxon test

	Maximum force (N)			Impulse (Ns)			Impulse duration (s)			Velocity (ms^−1^)			Effective mass (kg)		
	Hand	Chop	Fist	Hand	Chop	Fist	Hand	Chop	Fist	Hand	Chop	Fist	Hand	Chop	Fist
Dom.															
Mean	*2215*	*1864*	*1523*	15.02	12.35	*19.57*	*0.015*	*0.015*	0.026	*12.4*	11.6*	*7.7*	1.25	1.04*	2.597
St. dev.	776	697	446	6.49	4.18	6.63	0.004	0.001	0.005	2.4	2.4*	1.3	0.69	0.27*	0.86
Min	904	778	673	7.72	5.26	9.01	0.011	0.011	0.016	8.7	7.4	5.3	0.56	0.55	1.09
Max	4313	3809	2485	38.92	21.05	37.83	0.029	0.018	0.041	18.2	16.7	11.1	4.47	1.69	4.56
Non-dom.															
Mean	*1540*	*1512*	*1290*	12.68	10.97	*16.72*	*0.017*	*0.016*	0.027	*8.5*	*10.1*	*6.1*	1.53	1.09	2.756
St. dev.	551	699	646	5.50	3.94	6.72	0.004	0.003	0.007	1.3	2.2	1.0	0.74	0.33	1.02
Min	674	347	495	5.79	4.32	6.36	0.012	0.012	0.014	5.6	6.7	4.4	0.77	0.39	0.80
Max	3605	3402	4639	35.29	18.90	37.00	0.033	0.028	0.049	12.5	16.7	8.3	4.77	2.03	5.20

**Table 3:** The overview of punch parameters of the male (*n* = 29) and female (*n* = 21) subsamples. Italicized values are the mean values of parameters that differed significantly between the male and the female subsamples as indicated by the Mann-Whitney test. In 5 male volunteers, the velocity of the karate chop with the dominant hand could not be measured; thus, *n* = 24 for the parameters velocity and effective mass in the male subsample; the corresponding values are marked by an asterisk

	Maximum force (N)			Impulse (Ns)			Impulse duration (s)			Velocity (ms^−1^)			Effective mass (kg)		
	Hand	Chop	Fist	Hand	Chop	Fist	Hand	Chop	Fist	Hand	Chop	Fist	Hand	Chop	Fist
Male (*n* = 29)															
Dom.															
Mean	*2641*	*2192*	*1665*	*17.95*	14.995	22.84	0.015	0.015	0.027	13.1	12.7*	8.0	1.43	1.21*	2.93
St. dev.	682	633	401	5.53	3.08	5.73	0.004	0.001	0.005	2.3	2.3*	1.2	0.67	0.24*	0.81
Min	1560	1152	932	9.60	8.24	10.49	0.011	0.013	0.017	8.7	8.3	5.7	0.82	0.87	1.15
Max	4313	3809	2485	38.92	21.05	37.83	0.029	0.018	0.041	18.2	16.7	11.1	4.47	1.69	4.56
Non-dom.															
Mean	*1806*	*1811*	*1506*	14.72	*13.38*	20.16	0.017	0.016	0.029	8.8	10.8	6.2	1.72	1.27	3.27
St. dev.	525	600	714	5.01	2.90	6.25	0.005	0.002	0.007	1.3	2.4	0.9	0.73	0.27	0.93
Min	937	1002	688	8.69	7.78	10.09	0.013	0.013	0.018	6.7	6.7	4.4	0.87	0.89	1.77
Max	3605	3188	4639	35.29	18.90	37.00	0.033	0.023	0.049	12.5	16.7	8.3	4.77	2.03	5.20
Female (*n* = 21)															
Dom.															
Mean	*1628*	*1412*	*1326*	*10.98*	8.76	15.07	0.014	0.014	0.025	11.5	10.4	7.3	1.00	0.84	2.12
St. dev.	421	489	430	5.42	2.37	4.82	0.004	0.001	0.005	2.1	1.8	1.2	0.64	0.15	0.67
Min	904	778	673	7.72	5.26	9.01	0.011	0.011	0.017	8.7	7.4	5.3	0.56	0.55	1.09
Max	2423	2629	1959	33.41	16.88	30.51	0.027	0.016	0.04	0	14.3	9.5	3.67	1.18	3.51
Non-dom.															
Mean	*1173*	*1099*	*992*	9.87	*7.65*	12.00	0.017	0.016	0.024	8.2	9.3	6.0	1.25	0.83	2.03
St. dev.	318	605	351	4.84	2.42	3.64	0.004	0.004	0.004	1.2	1.5	1.0	0.68	0.21	0.61
Min	674	347	495	5.79	4.32	6.36	0.012	0.012	0.014	5.6	7.4	4.7	0.77	0.39	0.80
Max	1933	3402	1909	29.69	16.14	20.56	0.026	0.028	0.036	10.5	12.5	8.3	4.01	1.30	3.03

**Fig. 2:**
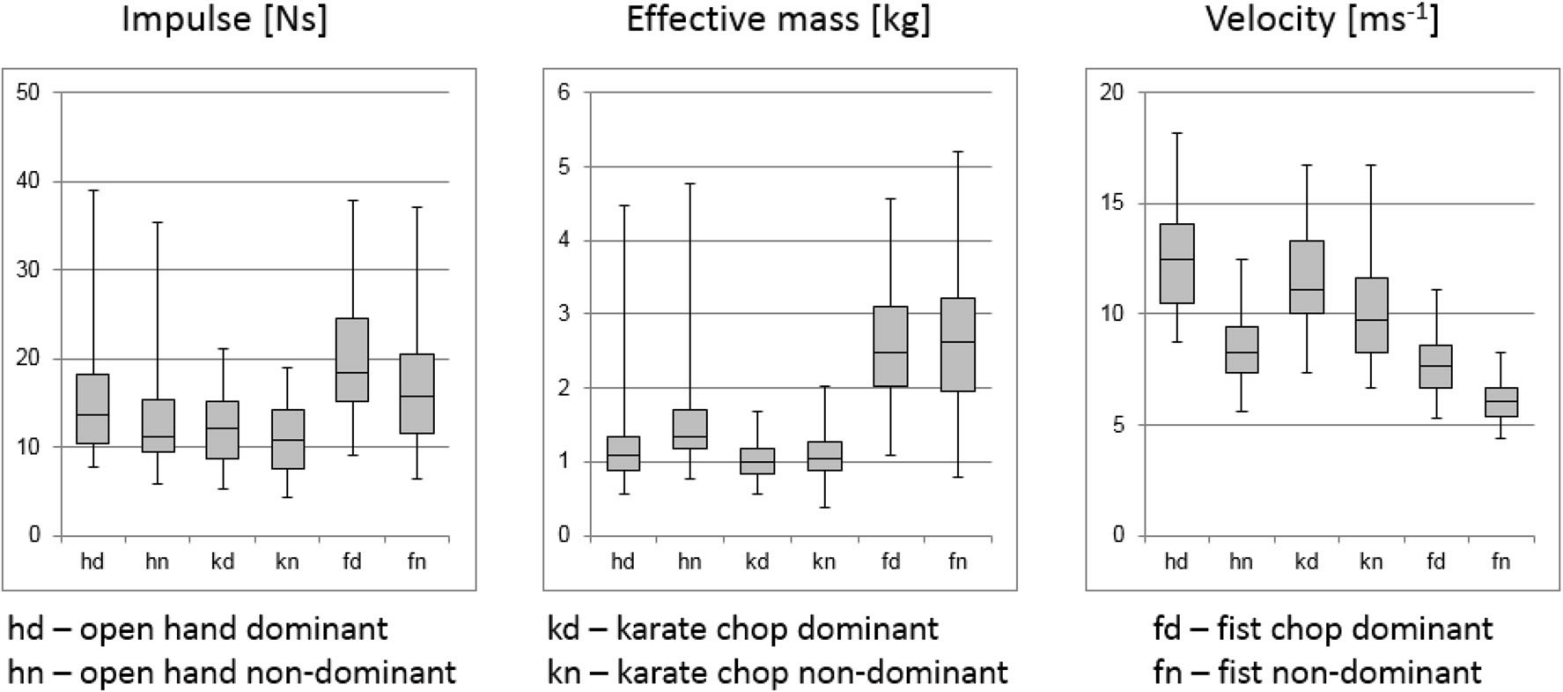
Boxplots of the most important punch parameters for the dominant and non-dominant hands

**Fig. 3:**
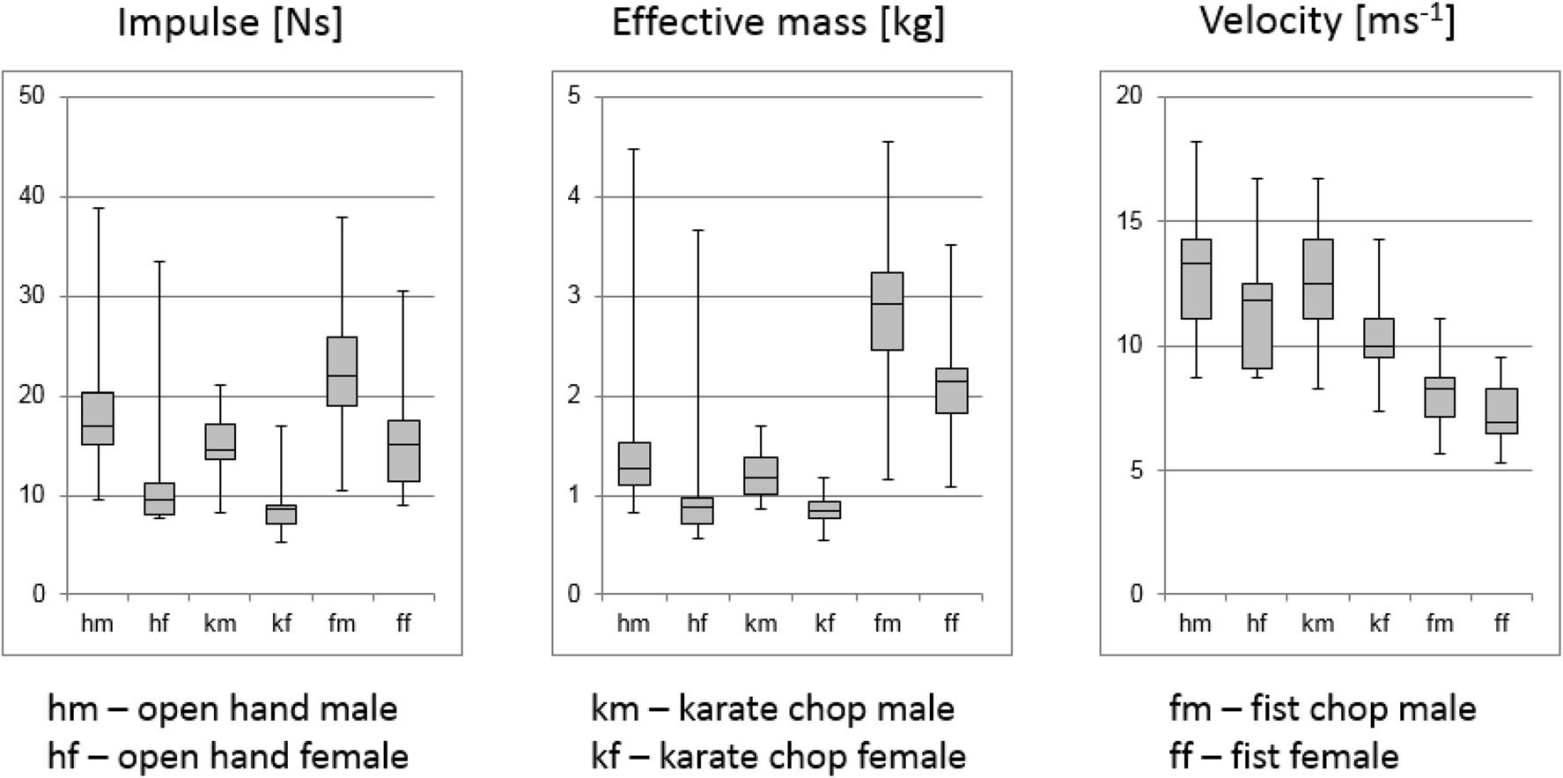
Boxplots of the most important punch parameters for the male and female subsamples

Figures 2 and 3 give a graphic overview of the parameter distribution and allow a comparison between dominant and non-dominant hands (Fig. 2) and between male and female volunteers (Fig. 3).

The Friedman test showed significant differences among the different punch types for all the parameters on a 0.05 significance level. The post hoc testing, according to the Nemenyi method, revealed that the fist punch parameters differed from the ones of the two other punch types in both the dominant and non-dominant hands. A significant difference between the palm strike and the karate chop showed for the maximum force and the impulse of the dominant hand and for the impulse, velocity, and effective mass of the non-dominant hand.

**Fig. 4:**
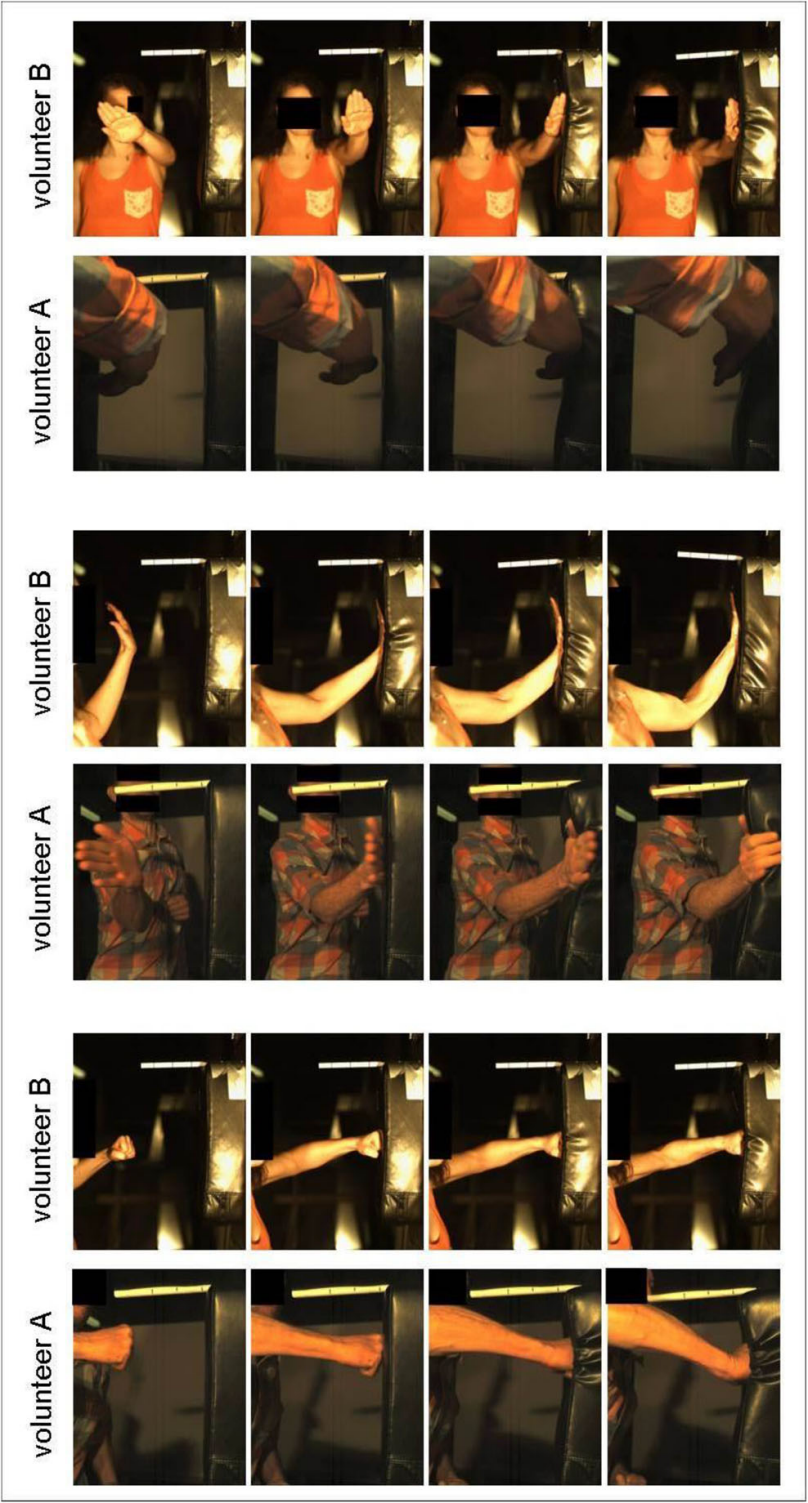
The final movement sequence of the punching movement of volunteers A and B. Top: karate chop. Center: palm strike. Bottom: fist punch

**Fig. 5:**
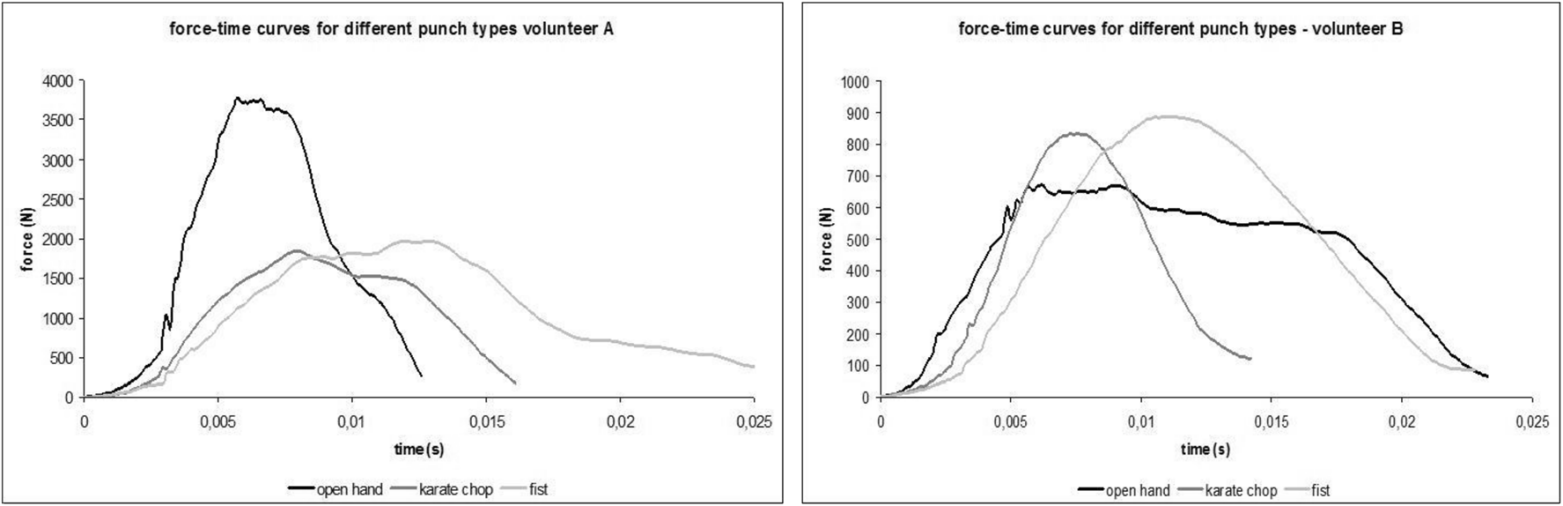
The force-time curves for the punches depicted in Fig. 4. Please note the different scales on the vertical (force) axis

An example of the final phase of the hand movement and the respective force-time curves for the three punch types is shown in Figs. 4 and 5. The striking difference between the volunteers—the long duration of the palm strike in volunteer B as compared with that in volunteer A—is based on the striking technique. For most volunteers, the impact duration was the longest for the fist punch and the shortest for the palm strike.

The Spearman correlation showed a statistically significant relationship between the body height and the impulse/effective mass and even stronger between the body weight and these parameters for all punch types; the correlation coefficient values ranged between 0.39 and 0.80 (see Table 4).

**Table 4:** Spearman correlation between body height/body weight and the impulse/effective mass for different punching techniques

	Fist palm				Karate chop				Palm strike			
	Dom.		Non-dom.		Dom.		Non-dom.		Dom.		Non-dom.	
	*I*	*m*_eff_	*I*	*m*_eff_	*I*	*m*_eff_	*I*	*m*_eff_	*I*	*m*_eff_	*I*	*m*_eff_
B. H.	0.60	0.46	0.48	0.49	0.55	0.67	0.51	0.41	0.55	0.45	0.53	0.39
B. W.	0.78	0.76	0.68	0.73	0.64	0.80	0.67	0.60	0.68	0.62	0.68	0.5

## Discussion

The presented data constitute a solid basis for the comparison of the most relevant physical parameters of different punching techniques and the understanding of punch dynamics under various circumstances. However, the reader should realize that it is not possible to relate the measured quantitative parameters directly to the known biomechanical tolerance values of various tissues of the human head in real-world forensic analyses.

The padding—necessary for the sake of volunteer safety—has naturally modified the impact and thus the dynamic parameters obtained in the lab cannot be viewed as applicable to punches between the hand and the head/face of a person. Whereas the punch velocity should not be affected at all and the impulse and effective mass presumably only to a small extent (considering a comparable situation with supported head, see below), the maximum force and the impact duration on the other hand were definitely altered by the padding—in punches against the pad, longer impacts and lower force amplitudes are expected than if they would have been registered in punches against the head (i.e., a sturdy bone structure covered by a thin deformable layer of the skin).

Regarding the injury risk associated with punches against the head, the impulse in combination with the surface area (i.e., the form) and the rigidity of the impactor are crucial. A large-area contact leads to lower stresses in the affected tissues and thus a lower injury risk for contact injuries. The same amount of (maximum) force in a palm strike with the whole palm and finger area contacting the head leads to a significantly lower injury risk than in a fist punch of the same force amplitude (with force being transmitted solely through the knuckles of the basis of the long fingers). The small contact area, the high rigidity of the impactor, and the high impulse of the fist punch make this punch type the most effective/dangerous as expected.

In accordance with our previous findings in a study dealing with slaps in different graduations [10], this study revealed that most subjects performed the palm strike not as a punch with the contact force evenly distributed on the palm and the volar aspects of the fingers (as is the case with slaps performed as “symbolic” punches). However, they hit the boxing pad primarily with the lower part of the hand/the wrist region. This lowers the area of (severe) contact and increases the effective mass and thus makes the palm strike more powerful—and more dangerous—than one might intuitively expect. Our data suggest in consideration of the biomechanical tolerance of the skull [11–16] that facial fractures and other injuries could easily result from such punches. The analysis of the casework at the Institute of Legal Medicine of the Munich University revealed that such injuries do occur after palm strikes, even though not frequently [17].

As it is a well-known fact, the injury risk of a punch depends to a high degree in the assailant`s technical skills. There seems to be little improvement in the punch velocity by training [18]. It appears that the impact force and thus the injury risk are higher in skilled persons because of them linking more of the mass of the striking arm into the punch [9, 18].

Another factor influencing the punch kinetics to a high degree is the support (or the lack thereof) of the target. In punches against a free moving, initially stationary, or moving head, it would have been accelerated in the direction of the punch and thus the impact energy transformed in both deformation and kinematic energy. Our test setup allowed only for deformation and corresponded thus to a situation with restrained head movement (head placed on the ground or leaned against a wall or strikes downwards against the top of the head supported by the spine). Situations of this kind lead to a higher impact force compared with free moving head. Introducing a mass scaling factor as a ratio between the head mass (approximately 4.5 kg) and the mass of the impacting body part [19] estimated a more than fourfold increase of the mean impact force for supported head in stomping based on the assumption of 15 kg impact mass of the foot/leg complex; using the same procedure and an effective impact mass of 1–4 kg, an increase of 20–90% of the mean contact force can be derived for punches as a rough estimate.

The results exhibit a considerable amount of interindividual variability in the punch parameters. The data show a significant relationship between the punch severity and the anthropometrical characteristics, especially between the effective mass and the resulting impulse and effective mass of the punch; the correlation coefficients ranged between 0.5 and 0.80 and thus, the relationship appears to be as tight as the one between the body height and the body weight (0.73 for our sample).

As expected, male subjects performed in general more severe punches (higher impulse) and the punches with the dominant hand were more effective than with the non-dominant hand for both male and female volunteers. However, the value ranges overlap, and the individual punching capabilities cannot be estimated simply based on the gender, punch type, dominant or non-dominant hand used etc.

Even though punches with the dominant hand are stronger and the volunteers felt much more comfortable punching with the dominant hand, the non-dominant hand can produce an impulse of comparable intensity; interestingly, the punch velocity was most times (much) lower with the non-dominant hand, but the effective mass (slightly) was higher suggesting generally a good punching technique of the non-dominant hand.

It is to note that in our sample only persons without special training (box, martial arts, etc.) were included. A long-term training of punching capabilities would presumably lead to a significant increase in punch severity [20, 21]; this particular issue will be addressed in our future study; common sense as well as the known literature data suggests that special training is a critical factor determining punching capabilities (together with body mass).

## Limitations

The target (the punching pad) height was predefined and thus could not be chosen freely by each volunteer. However, its size (target surface approx. 40 × 20 cm) and placement with its long side vertically allowed for individual tuning of the punching motion.

As opposed to a punch against an opponent, the measurement setup was such as apart from the padding there was no yield of the target (no movements of the target due to the impact force). This might have influenced the punch perception and thus the performance of the volunteers. However, the setup was the same for all volunteers and all covered punch scenarios so that the results are comparable. Moreover, the measurements started only after each participant warmed up and felt comfortable carrying out punches against the pad.
